# Systematic identification of genes involved in divergent skeletal muscle growth rates of broiler and layer chickens

**DOI:** 10.1186/1471-2164-10-87

**Published:** 2009-02-22

**Authors:** Qi Zheng, Yong Zhang, Ying Chen, Ning Yang, Xiu-Jie Wang, Dahai Zhu

**Affiliations:** 1 State Key Laboratory of Plant Genomics, Institute of Genetics and Developmental Biology, Chinese Academy of Sciences, Datun Road, Beijing, PR China; 2 National Laboratory of Medical Molecular Biology, Institute of Basic Medical Sciences, Chinese Academy of Medical Sciences and Peking Union Medical College, 5 Dong Dan San Tiao, Beijing, PR China; 3 Department of Animal Genetics and Breeding, College of Animal Science and Technology, China Agricultural University, 2 Yuanmingyuan West Road, Beijing, PR China; 4 Graduate University of the Chinese Academy of Sciences, Beijing, PR China

## Abstract

**Background:**

The genetic closeness and divergent muscle growth rates of broilers and layers make them great models for myogenesis study. In order to discover the molecular mechanisms determining the divergent muscle growth rates and muscle mass control in different chicken lines, we systematically identified differentially expressed genes between broiler and layer skeletal muscle cells during different developmental stages by microarray hybridization experiment.

**Results:**

Taken together, 543 differentially expressed genes were identified between broilers and layers across different developmental stages. We found that differential regulation of slow-type muscle gene expression, satellite cell proliferation and differentiation, protein degradation rate and genes in some metabolic pathways could give great contributions to the divergent muscle growth rates of the two chicken lines. Interestingly, the expression profiles of a few differentially expressed genes were positively or negatively correlated with the growth rates of broilers and layers, indicating that those genes may function in regulating muscle growth during development.

**Conclusion:**

The multiple muscle cell growth regulatory processes identified by our study implied that complicated molecular networks involved in the regulation of chicken muscle growth. These findings will not only offer genetic information for identifying candidate genes for chicken breeding, but also provide new clues for deciphering mechanisms underlining muscle development in vertebrates.

## Background

Myogenesis is a very complicated but precisely regulated developmental process whose mal-regulation can cause many neuronal, heart and muscular dysfunction diseases. During the embryonic period, muscle development starts with the emergence of somites. Myogenic precursor cells originated from somites first give rise to myoblasts, which will further undergo proliferation, migrate to their final locations and fuse into multinucleated myotubes, finally differentiate into mature muscle fibres [1]. In general, the final number of muscle fibres is fixed at the end of embryogenesis [2]. Muscle growth after birth is achieved by increase of fibre sizes, which is the consequence of satellite cells fusion to existing fibres [3,4]. Mature muscle fibres exhibit a remarkable plasticity, in response to external stimuli or disease. Physical exercise and mechanical loading are capable of producing significant increase in muscle mass by muscle fibre hypertrophy [5-7]. In contrast, during many disease states, such as cancer, AIDS and diabetes, a dramatic loss of skeletal muscle mass is observed [8]. The broiler (chicken for meat production) and layer (chicken for egg production) chickens are ideal model systems to study the controlling mechanisms of myogenesis rate and muscle cell size. During the past 80 years, genetic selection has been concentrated on high growth velocity and large muscle mass for broilers; in contrast, layers have been selected for egg production. Therefore, even under optimal growth conditions, the body size of layers is still much smaller than that of broilers due to their intrinsic genetic differences. Such unique biological feature of broilers and layers allows us to investigate many interesting questions related to muscle growth control. For example, what makes chicken skeletal muscle fibres grow two or three times faster in broilers than in layers? What is the molecular genetic basis for such growth difference? Due to the high homology of chicken and human protein coding genes, answers to above questions will also shed light on studies of human muscle development regulation.

Many researchers have made great efforts to investigate the divergent muscle growth rates and muscle cell sizes of broilers and layers and attempted to decipher the underlining mechanisms of above phenomena. Some previously published works observed that broilers have higher food intake [9], less activity [10] and lower heat production [11,12] than layers. Broilers also have more muscle fibre cells and larger fibre sizes than layers [13,14]. From the physiological point of view, the neuroendocrine system is an indispensable factor to regulate animal growth. Zhao R. *et al*. found the hypothalamic somatostatin (*SS*) mRNA expression was higher in layers than in broilers whereas the expression of hepatic GH receptor (*GHR*) mRNA was opposite [15]. Cassy S. *et al*. also reported that broilers were less sensitive or responsive to peripheral concentrations of leptin than layers [16]. Several studies demonstrated that the overall whole-body protein turnover rate is lower in broilers compared to layers [17,18]. A few published data showed that layers have higher calpain activity and lower calpastatin activity than broilers [19,20]. At the genetic level, QTL mapping analysis also identified several dozens of QTLs associated with the body mass trait of different chicken breeds on multiple chromosomes [21-23].

Despite these progresses, genome-wide systematic study for the genetic basis and molecular mechanisms implicated in the divergent muscle growth rates between broilers and layers is still lacking. In the present study, we used Affymetrix chicken genome array to systematically identify differentially expressed genes in skeletal muscle tissues between broilers and layers at five posthatch developmental stages. A total of 543 differentially expressed genes were identified between broilers and layers. Functional analysis showed that muscle development related GO terms were enriched among differentially expressed genes. Greater expression ratio of type II to type I fiber genes and more activity of genes related to satellite cell proliferation and differentiation were observed in broilers than in layers, which could be important factors contributing to the divergent muscle growth rates of the two chicken lines. We also identified a few genes whose expression profiles were highly correlated with the growth rates of broilers and layers, and found several metabolic pathways significantly over-presented among differentially expressed genes in broilers and layers.

## Results

### Differentially expressed genes between broilers and layers

Broiler and layer chickens showed significantly divergent skeletal muscle growth rates during development, and the body weight increment difference between broilers and layers is most significant during posthatch 2–6 weeks (see Additional file 1). To identify the underlining molecular mechanisms of the above phenomena, we used Affymetrix Chicken Genome Array to examine the gene expression profiles of broiler and layer skeletal muscle cells at different developmental stages. RNA samples from broiler and layer skeletal muscle cells at posthatch 1 day (1 d), 2 weeks (2 w), 4 weeks (4 w), 6 weeks (6 w) and 8 weeks (8 w) were extracted and used in the microarray experiment. An average of about 41% chicken genes was detected to be expressed on each microarray. Statistical analysis showed that the signal concordance of three technical replicates at each time point was very high (R^2 ^> 99.8%), indicating that our data contained very limited artificial variance.

**Figure 1:**
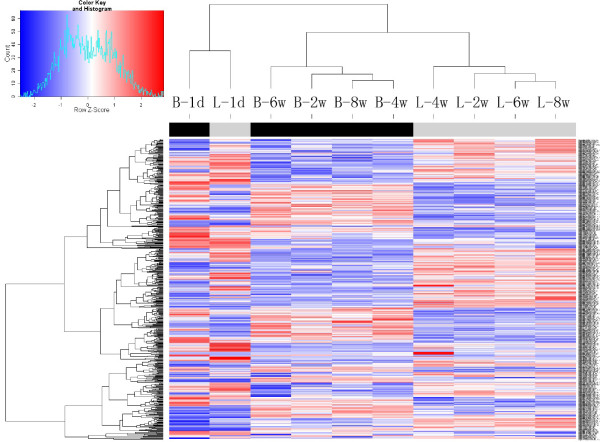
**Heat map of differentially expressed probe sets between broilers and layers across different developmental stages**. Columns marked black at top represent samples from broilers and columns marked light gray at top represent samples from layers. The small figure represents color scales used in the heat map.

Using ANOVA method, we identified 543 genes that were significantly differentially expressed (DE) with changes ≥ 1.5 fold and p-value < 0.01 between broilers and layers considering all examined time points. The estimated False Discovery Rate (FDR) of DE genes was 0.02%. Among the 543 genes, 366 are known genes and the rest 177 are unknown genes, including ESTs and hypothetical ORFs (Additional file 2). The clustering result demonstrated that majority of DE genes exhibited opposite expression patterns in broilers and layers (Figure 1). Except for post-hatch 1 d, the expression patterns of the 543 genes were more correlated among samples at different time points of the same chicken line than between different chicken lines.

To confirm the microarray hybridization results, quantitative real-time RT-PCR (qRT-PCR) was performed on 52 randomly selected DE genes. Among them, 46 (81%) genes exhibited expression patterns consistent with the microarray results (t-test, p < 0.01, see Additional file 3), indicating that our microarray hybridization results indeed reflect the relative *in vivo *expression level of each gene.

**Figure 2:**
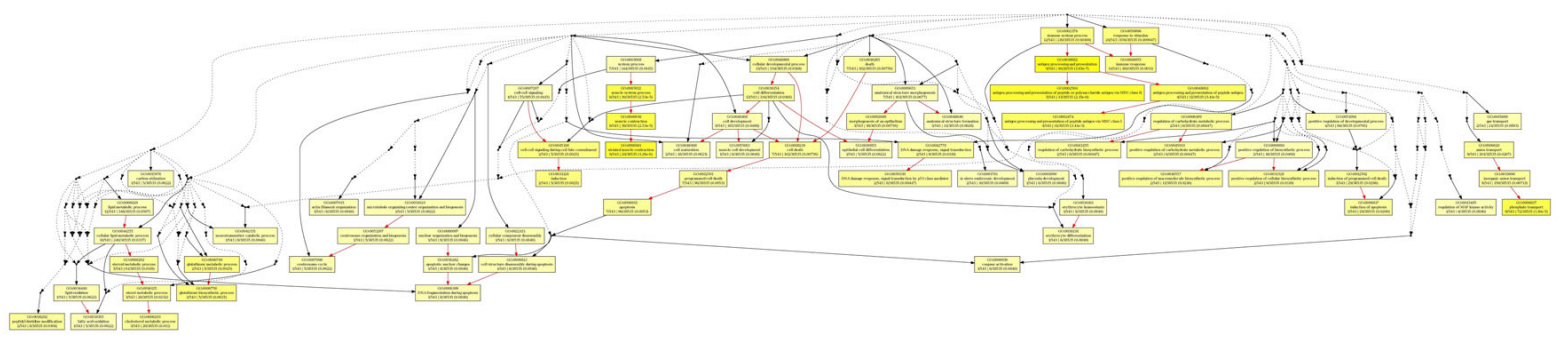
**Significantly enriched GO terms in the biological process category among DE genes**. Significantly enriched GO terms were represented in yellow boxes. The color saturation degrees positively correlate with the significant of enrichment. Black points represent non-significant GO nodes required for building up the dendrogram.

To elucidate the correlation between gene expression pattern and the skeletal muscle development difference of broilers and layers, we investigated the functional bias of these differentially expressed genes according to Gene Ontology classifications. As expected, majority of enriched GO terms among the differentially expressed genes between broilers and layers were muscle development and metabolic related (Figure 2 and see Additional file 4). Other enriched GO terms were related to cell development, apoptosis and some signalling pathways (Table 1). In addition, immune response related processes were also enriched among the differentially expressed genes. This observation is consistent with the previous report that the immune system of different chicken breeds is very divergent [24].

**Table 1:** List of differentially expressed genes related to muscle growth and development.

**Gene Title**	**Symbol**	Fold change*				
		
		**1 D**	**2 W**	**4 W**	**6 W**	**8 W**
**Slow-type myofiber protein genes**						
troponin I type 1 (skeletal, slow)	*TNNI1*	-1.99	1.05	-6.47	-1.05	1.14
Myoglobin	*MB*	1.04	-1.81	-10.04	-6.8	1.17
myosin, light chain 3, alkali; ventricular, skeletal, slow	*MYL3*	-1.96	-1.04	-19.79	-1.41	-1.02
myosin, heavy chain 7B, cardiac muscle, beta	*MYH7B*	-3.07	-1.01	-11.31	1.1	1.36
similar to myosin L2B regulatory light chain, cardiac muscle – chicken	LOC417506	-1.27	1.04	-58.02	-5.54	1.34
myelin basic protein	MBP	1.16	-1.81	-1.83	-3.67	-1.68
similar to dynein light chain-2	LOC417663	-1.25	-2.13	-1.77	-1.28	-1.73
**Satellite cell proliferation and muscle hypertrophy**						
cysteine and glycine-rich protein 3 (cardiac LIM protein)	*CSRP3*	-2.17	1.01	-9.44	-16.72	-1.04
four and a half LIM domains 2	*FHL2*	-1.38	-8.19	-10.95	-7.65	-9.84
similar to actin binding LIM protein family member 2	LOC422866	-1.08	-2.06	-1.78	-2.24	-2.56
fibroblast growth factor receptor 2	*FGFR2*	-1.31	-2.28	-1.05	-1.6	-1.87
fibroblast growth factor 1 (acidic)	*FGF1*	-1.11	-2.45	-2.07	-2.75	1.61
heparan sulfate 6-O-sulfotransferase 2	*HS6ST2*	-3.07	-4.09	-1.51	-2.13	-3.1
musculoskeletal, embryonic nuclear protein 1	*MUSTN1*	-1.01	2.94	2.17	2.45	1.51
fibroblast growth factor 16	*FGF16*	-1.06	2.86	2.45	8.16	2.77
inner centromere protein antigens 135/155 kDa	*INCENP*	2.02	1.59	2.15	1.39	1.3
nudE nuclear distribution gene E homolog 1 (A. nidulans)	*NDE1*	1.49	2.48	2.68	1.91	1.23

*Expression values of the broiler muscle cells are compared to those of the layer muscle cells, which had been normalized to a fold change of 1.0 when broilers have more mRNAs, or -1.0 when broilers have less.

### Expression difference of genes related to fast-type and slow-type fibres

**Figure 3:**
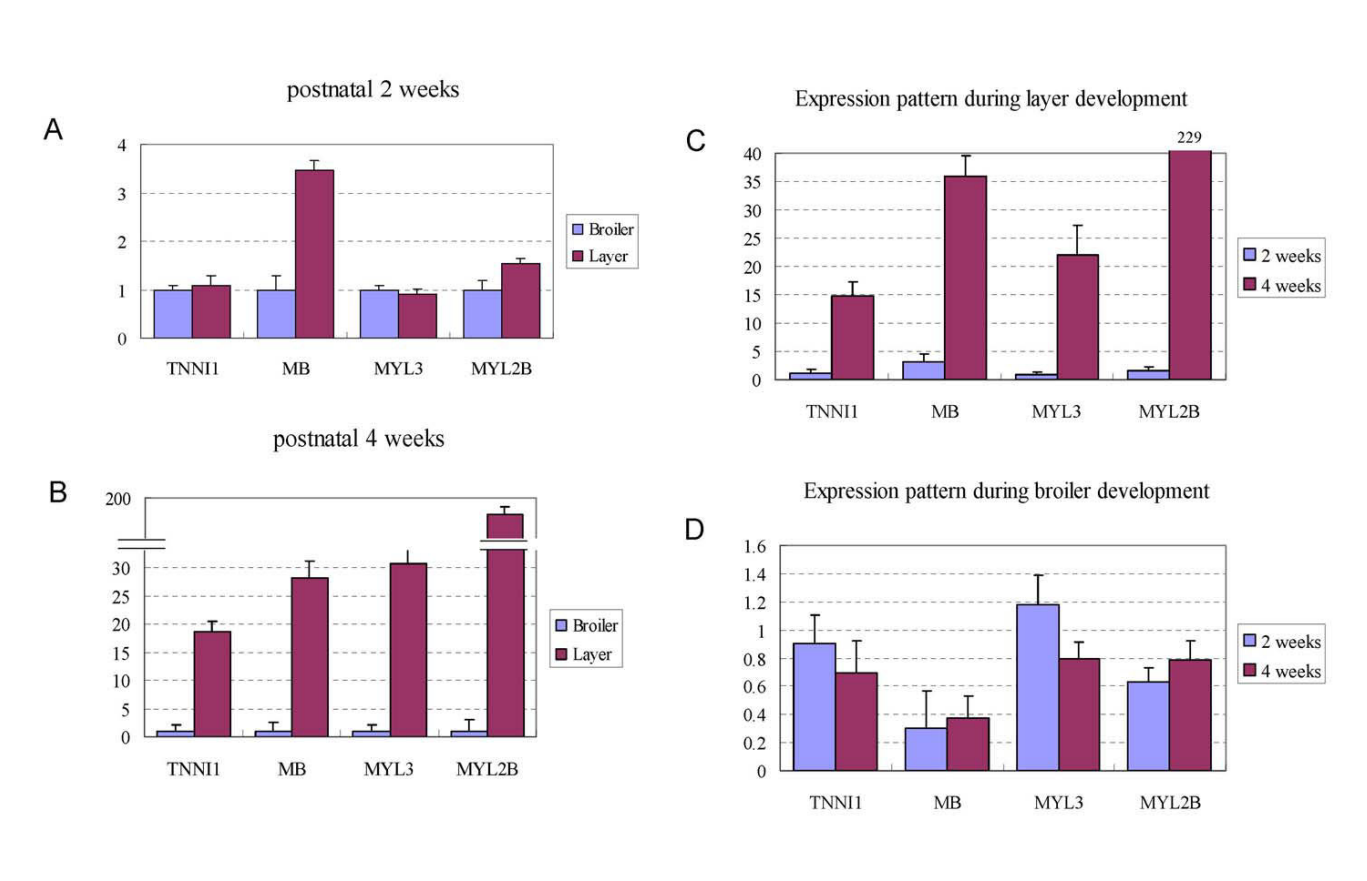
**qRT-PCR results of several slow-type fibre genes**. A. Expression comparison of four slow-fibre genes between broilers and layers at postnatal 2 weeks. The expression levels of four genes in layers shown in panel A and panel B are all compared to that in broilers which are all normalized to 1.0. B. Expression level comparison of four slow-fibre genes between broilers and layers at postnatal 4 weeks. C. The expression pattern of four genes during layer development (at 2 weeks and 4 weeks). D. The expression pattern of four genes during broiler development (at 2 weeks and 4 weeks).

Previous studies reported that selection for rapid growth increases the number and size of muscle fibres without changing their fibre type composition in chickens [13]. However, based on our array data, we found that the expression ratio of fast to slow-type muscle fibres is different in *Pectoralis Major *muscle cells between broilers and layers. The expression levels of several slow-type muscle protein encoding genes, troponin I type 1 (*TNNI1*), myoglobin (*MB*), myosin light chain 3 (*MYL3*), myosin heavy chain 7B (*MYH7B*) and myosin L2B regulatory light chain (*MYL2B*), were significantly higher in layers than in broilers (Table 1, Figure 3A and 3B), which could contribute to the slower growth rate of layer muscle cells than that of broilers.

In addition, the above slow-type muscle genes exhibited varied expression patterns during posthatch muscle development. Dramatically upregulated expressions of these slow-type genes were observed in layers at 4 weeks but not in broilers (Figure 3C and 3D), indicating that fast-slow switch of muscle fibres might occur in layers at posthatch 4 weeks, which is the most significant body weight increment period for broilers.

### Divergent regulation of satellite cell proliferation and differentiation

Like other vertebrate animals, posthatch muscle growth in chicken mainly results from muscle hypertrophy through satellite cell activation. Therefore, satellite cell proliferation and differentiation is a key regulatory factor for postnatal muscle growth and hypertrophy. The muscle fibre size of broilers is 2–3 times larger than that of layers, which prompts us to consider that genes involved in the satellite cell proliferation and differentiation could be potential regulators for the divergent muscle growth of broiler and layer chickens.

**Figure 4:**
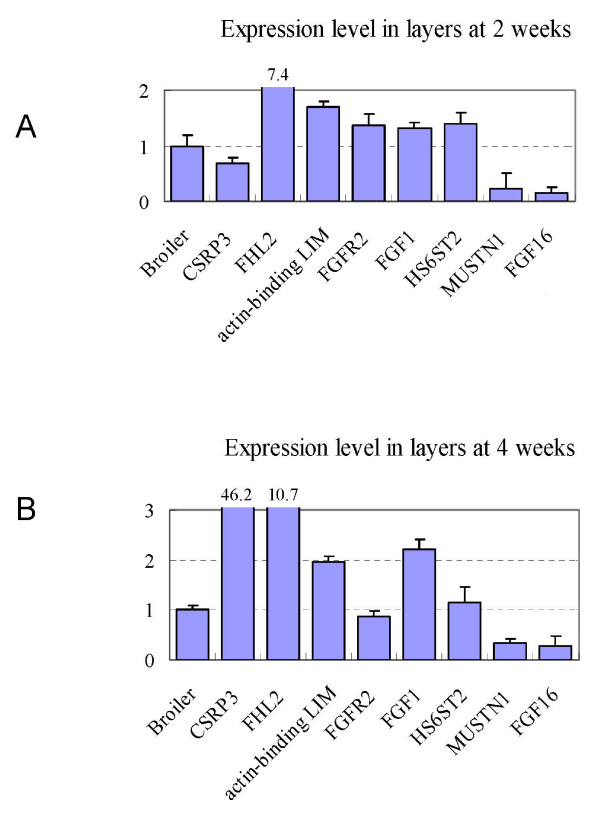
**qRT-PCR results of several satellite cell proliferation and muscle hypertrophy related genes**. The expression levels of various genes in layers are compared to that in broilers which were all normalized to 1.0.

As expected, we identified a few differentially expressed genes involved in satellite cell proliferation, differentiation and muscle hypertrophy (Table 2, Figure 4). Several LIM domain containing protein encoding genes were differentially expressed between broilers and layers. LIM proteins are involved in muscle development through mechanotransduction signalling processes to regulate gene expression [25,26]. Downregulation of LIM protein CSRP3 has been reported in human undergoing pathogenic cardiac hypertrophy [25]. Consistently, significantly lower level of CSRP3 transcripts was detected in broilers than in layers at posthatch 4 weeks, the rapid growth period of chicken, which may contribute to the larger muscle cell size of broilers.

**Table 2:** List of differentially expressed genes related to metabolic regulation.

**Gene Title**	**Symbol**	Fold change*				
		
		**1 D**	**2 W**	**4 W**	**6 W**	**8 W**
**Carbohydrate metabolism**						
pyruvate dehydrogenase complex, component X	RCJMB04_17g4	1.65	1.72	1.44	1.83	1.41
aldehyde dehydrogenase 1 family, member A2	*ALDH1A2*	2.09	1.33	1.39	1.5	1.33
Glycosyltransferase	AER61	2.29	1.61	1.94	2.23	1.75
aldehyde oxidase 1	AOX1	-1.64	2.05	1.52	2.07	2.2
UDP glycosyltransferase 8 (UDP-galactose ceramide galactosyltransferase)	UGT8	-1.05	-1.5	-1.76	-2.8	-1.58
**Fatty acid metabolism**						
ELOVL family member 6, elongation of long chain fatty acids (FEN1/Elo2, SUR4/Elo3-like, yeast)	RCJMB04_16d24	1.45	1.41	1.97	1.84	1.58
similar to intestinal 15 kda protein; FABP6	LOC416154	1.06	1.32	1.79	1.7	2.05
fatty acid binding protein 4, adipocyte	*FABP4*	-1.38	2.16	2.5	2.14	1.34
3-oxoacid CoA transferase 1	*OXCT1*	1.22	2.23	2.55	2.54	1.92
adiponectin receptor 2	ADIPOR2	-2.52	-1.11	-1.61	-2.12	-1.68
Thiolesterase B	LOC415786	-5.16	-1.75	-1.02	-1.24	1.1
sterol carrier protein-2	LOC396550	-1.49	-1.34	-1.27	-2.09	-2.52
**Regulation of glucose and fatty acid metabolism**						
pyruvate dehydrogenase kinase, isozyme 4	*PDK4*	-1.01	1.93	-3.88	-29.95	3.85
insulin induced gene 1	RCJMB04_1d1	1.13	1.92	2.02	3.38	1.3
**Hormone synthesis**						
catechol-O-methyltransferase	*COMT*	2.56	2.06	2.77	2.02	1.27
Hydroxysteroid (17-beta) dehydrogenase 7	HSD17B7	1.1	1.51	1.68	2.28	1.24
**Ubiquitin-mediated protein degradation**						
hect domain and RLD 4	*HERC4*	-2.19	-1.77	-2.72	-1.86	-1.87
similar to hect domain and RLD 5; cyclin-E binding protein 1	LOC422513	-1.02	-1.03	-8.77	-1.71	1.07
ring finger protein 12	*RNF12*	-2.24	-1.76	-1.45	-1.8	-1.67
F-box protein 22	RCJMB04_6j20	1.08	-2.2	-3.61	-2.24	1.21

*Expression values of broiler muscle cells are compared to the layer muscle, which had been normalized to a fold change of 1.0 when broiler has more of the mRNA, or -1.0 when broiler has less.

FHL2 was primarily identified as a LIM domain protein down-regulated in rhabdomyosarcomas [27]. Previous observation that stable expression of FHL2 in mouse myoblasts induced differentiation of myoblasts into myotubes [28] suggested that chicken FHL2 might be a regulator of muscle growth and hypertrophy through controlling satellite cell proliferation and differentiation. We also observed that the expression level of *fhl2 *is lower in broilers than in layers.

FGF2 is a potent stimulator of myoblast and satellite cell proliferation, and an intense inhibitor of cell differentiation as well [29]. The FGF signalling pathways participate in heparin sulfate (HS) modification of heparan sulfate proteoglycans (HSPGs) [30,31]. Another protein, HS6ST2, involves in the modification of heparin sulfate (HS) biosynthesis. We found that the expression level of *FGFR2 *and *HS6ST2 *in layers was higher than in broilers, although the expression of *FGF2 *did not show significant difference. Such enhanced FGF2 signalling in layers could inhibit satellite cell differentiation to influence muscle growth and hypertrophy.

*MUSTN1 *is a muscle development related gene. It has been reported that exercises increased the expression level of *MUSTN1 *in hypertrophic muscle [32]. We also observed a higher expression level of *MUSTN1 *in broilers than in layers, indicating that *MUSTN1 *plays a role in skeletal muscles hypertrophy regulation.

### Differentially expressed metabolic enzymes between broilers and layers

**Figure 5:**
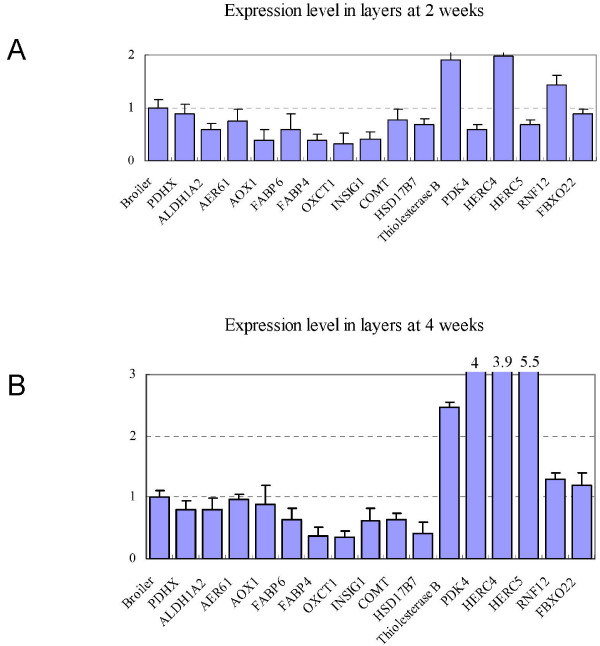
**qRT-PCR results of genes encoding some metabolic enzymes and regulators**. The expression levels of selected genes in layers are compared to that in broilers which were all normalized to 1.0.

Difference in metabolic rates could be another factor causing the body weight divergence of broilers and layers. Several metabolic related genes were found to be differentially expressed between broilers and layers from the microarray data, including genes involved in glucose and glycogen metabolism, fatty acid transportation and utilization, and protein degradation (Table 2 and Figure 5).

Some glucose synthesis accelerating enzyme encoding genes, *PDHX*, *ALDH1A2*, *AER61 *and *AOX1*, had higher expression in broilers than in layers, whereas a fat break-down related enzyme thiolesterase B expressed higher in layers than in broilers, suggested that different metabolic regulation might be another contributor to the body weight difference. Several fatty acid transportation and utilization related genes had significant expression difference between broilers and layers, including genes encoding fatty acid binding protein 4 (*FABP4*), intestinal 15 kDa protein (*FABP6*) homolog, and elongation of long chain fatty acids (ELOVL family member 6). Genetic variation in bovine *FABP4 *gene has been found to be associated with intramuscular fat content and subcutaneous fat depth [33]. In our study, the expression levels of *FABP4 *and *FABP6 *were higher in broilers than in layers, in agreement with more intramuscular fat deposition of broilers.

Other two interesting differentially expressed genes were pyruvate dehydrogenase kinase 4 (*PDK4*) and 3-oxoacid CoA transferase 1 (*OXCT1*), which play important regulatory roles in glucose and fatty acid metabolic networks [34,35]. Catechol-O-methyltransferase (*COMT*) was another metabolic related gene. It has been shown that inhibition of *COMT *expression resulted in body weight lose in human [36]. Similarly, we observed a lower expression level of *COMT *in layers than in broilers. Notably, the Tyrosine metabolism and synthesis pathway and degradation of ketone bodies pathway were found significantly enriched of differentially expressed genes (Additional file 5), in which OXCT1 and COMT serve as key regulatory enzymes. Hydroxysteroid (17-beta) dehydrogenase 7 (*HSD17B7*) is a key enzyme for testosterone synthesis. Testosterone has pronounced effects on muscle protein synthesis and muscle mass enlargement, especially during the period of rapid muscle cell growth [37]. Higher expression level of *HSD17B7 *was observed in broilers, which might result in higher testosterone level thereby contributed to fast muscle growth and hypertrophy.

Ubiquitin mediated proteolysis regulates protein abundance and serves as a central regulatory function in many biological processes. It has been shown that the protein degradation rate in broilers is lower than in layers during muscle development [17,18]. However, whether such protein degradation difference is caused by ubiquitin mediated pathways remains elusive. Based on our microarray and qRT-PCR results, we found that genes encoding several classes of ubiquitin related proteins showed higher expression levels in layers than in broilers (Figure 5), including F box protein genes *FBXO22 *and *FBXO30*, ubiquitin carboxyl terminal hydrolase isozyme L1 (*UCH L1*), as well as several other E3 ligases (hect domain and RLD 4, *HERC4*; hect domain and RLD 5, *HERC5*; and ring finger protein 12, *RNF12*). Such overall lower expression of ubiquitin related protein degradation genes in broilers than in layers might reflect the lower degradation rate of skeletal muscle proteins in broilers, thereby contributing to the larger body sizes of broilers.

### Identification of QTE genes involved in muscle growth control

Because the growth rate of skeletal muscle cells vary a lot during development between broilers and layers, we wanted to identify genes with expression traits significantly correlated with chicken muscle cell growth rate, these genes might directly or indirectly involve in muscle cell growth control. Because the skeletal muscle tissue contributes the majority body weight of chicken [38], we used whole body mass of individual chickens to represent their skeletal muscle mass in this study. We examined the relationship between the expression value of each detected gene and the natural increase rate (NIR) of chicken body mass (Δg/g per day). NIR instead of absolute growth rate was used here because the same amount of absolute growth rate could be contributed by different NIRs with different initial body weights, thus should have different gene regulation patterns. As a result, 1,205 and 1,187 genes were found with expression patterns significantly correlated with the NIR of body mass of broilers or layers, respectively, with a overlap of 115 genes. These genes could be recognized as potential Quantitative Trait Expression (QTE) genes in chickens (see Additional file 6).

We also calculated the FDRs of QTE results by similar random permutation methods used for DE genes. As a result, the FDRs were 4.4%, 4.8% and 0.18% for identified QTE genes in broilers, layers and common genes between them, respectively.

**Figure 6:**
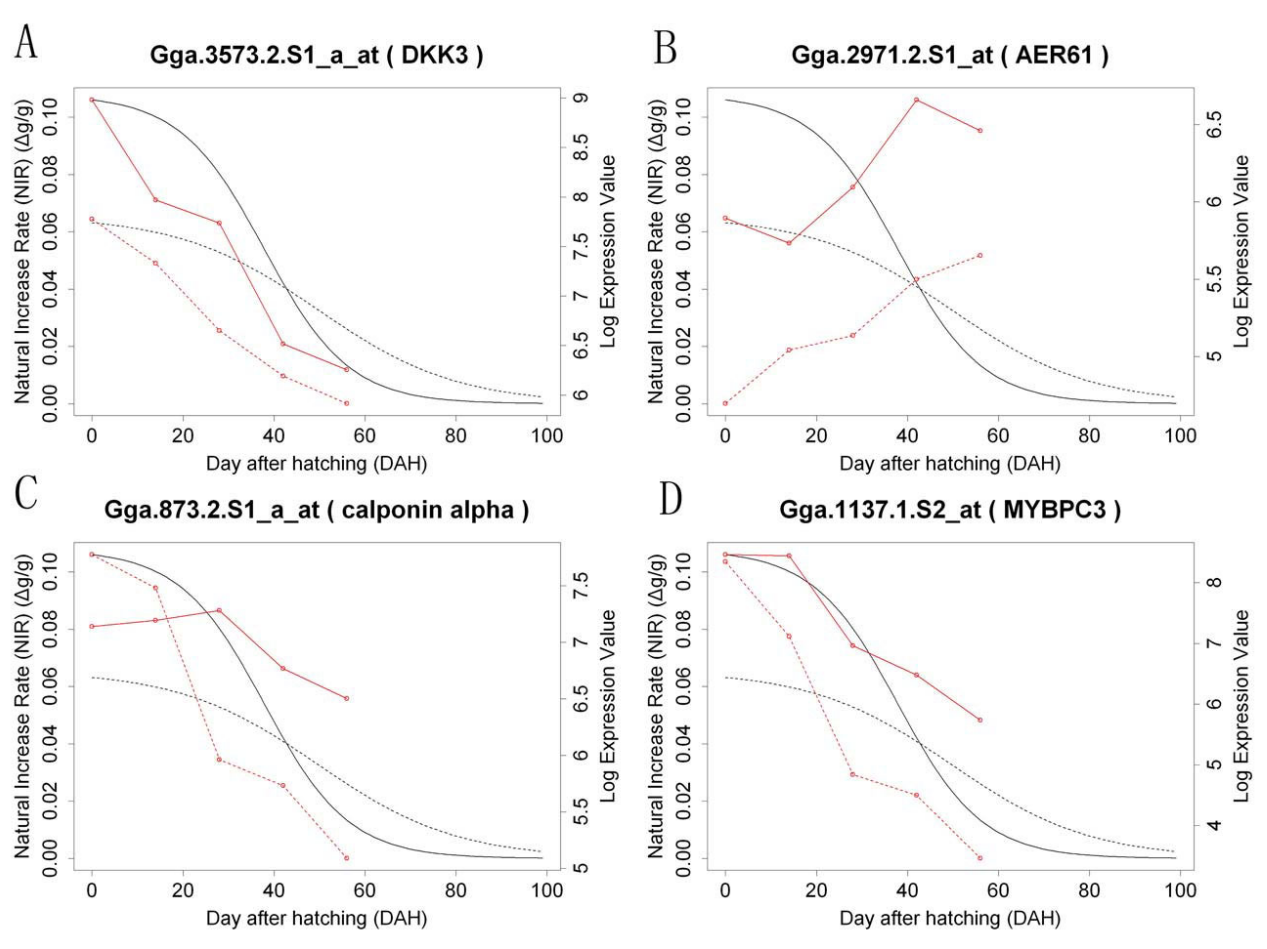
**Correlation of gene expression profiles and growth rates in broilers and layers**. The NIR of broiler chickens is shown as solid black curve, the NIR of layer chickens is shown as dashed black curve. The expression pattern of genes in broilers is shown as solid red curve, the expression pattern of genes in layers is shown as dashed red curve.

GO enrichment analysis results showed that the enriched GO terms among the common QTE genes were very different from that of the DE genes (see Additional file 7). Only few biological processes, such as muscle-related processes, could be found to be enriched both in the common QTE genes and the DE genes. Many developmental related processes and signal transduction processes were found to be enriched in the QTE genes, suggesting that a temporal regulatory mechanism would exist in chicken muscle development (see Additional file 8 and 9). Interestingly, our data suggested that the dickkopf homolog 3 (*DKK3*) gene could be a potential QTE gene both in broilers and layers. *DKK3 *is a tumour suppressor which could inhibit proliferation of many cancer cells [39-41]. Although there was no report on the function of *DKK3 *in muscle development regulation, we found it expressed at a relative high level in skeletal muscle cells of both chicken lines. In addition, significantly higher expression level of *DDK3 *in broilers than in layers was observed (Figure 6A), suggesting that it might contribute to the divergent muscle growth rates between broilers and layers. Several other genes with expression profiles correlated or reverse-correlated well with the growth rates of broilers and layers were also shown (Figure 6).

### Analysis of pathways and regulatory networks for chicken muscle development

The development of chicken muscle cells requires precise and coordinated regulation of many genes. As shown above, various pathways might involve in this regulatory network. To explore potential key regulators, we mapped DE genes to pathways collected by the KEGG database [42-44] and found a few metabolic pathways enriched of DE genes (see Additional file 10), including lipid metabolism, amino acid metabolism and nitrogen metabolism pathways. Interestingly, several cofactor and vitamin metabolism pathways were found to be enriched of DE genes, including vitamin B6, thiamine (vitamin B1) and glutathione metabolism pathways, suggested that vitamin-related nutrition metabolism might involve in chicken myogenesis. The DE genes in the glutathione (GSH) metabolism pathway included glutathione S-transferase kappa 1 (GSTK1), glutathione transferase zeta 1 (GSTZ1, or maleylacetoacetate isomerase, MAAI) and glutamate-cysteine ligase, catalytic subunit (GCLC). GCL (glutamate cysteine ligase) is the rate-limiting enzyme of GSH synthesis process, and plays pronounced regulatory role in neuron system. Although it is unknown whether GCL functions in muscle development, the catalytic subunit GCLC was reported to be activated by insulin through PI3K/Akt/mTOR/Nrf2/GCLC pathway upon hyperglycemia-induced stress in human brain endothelial cells, whereby attenuating the hyperglycemia-induced apoptosis via maintaining cellular redox balance [45]. As it has been observed that broilers are insulin and hyperglycemia resistant [46], we proposed that GCLC would be a potential regulator in hyperglycemia resistance process for broiler chickens.

## Discussion

The genetic closeness and divergent muscle growth rates of broilers and layers make them great models for myogenesis study. At the time of hatch, the process of myofibre formation is nearly complete and the numbers of myofibres are fixed in different chicken breeds [2]. The postnatal growth of myofibres is mainly through the enlargement of cell size, a process also called hypertrophy. It has been shown that the sizes of myofibres are much larger in broilers than in layers. Although there have been several reports studying the physiological differences of skeletal muscle cells in broilers and layers, very limited knowledge is known about the underlining molecular mechanisms. In this work, we reported the transcriptome comparison of broiler and layer breast muscle cells at different developmental stages, and identified many genes and processes that might contribute to muscle hypertrophy and muscle mass control.

The skeletal muscle RNA samples used in this study expanded from posthatch 1 d to 8 w, which covered the period with most divergent muscle growth rates. The growth rates of both broilers and layers slowed down after posthatch 8 w. As expected, many muscle structure and development related genes were differentially expressed between broilers and layers. It is worth to note that genes encoding several slow-type muscle proteins had lower expression in broilers than in layers at 4 weeks, which is consistent with previous studies that more fast-type fibres (especially type-IIB) were accumulated in broilers than in layers. In general, type-II fibres have larger diameter and are easier to response to various stress induced muscle hypertrophy. Accumulation of type-II fibres might be a major contributor to the fast growth rate of broilers at 4 weeks. (Table 2).

The postnatal muscle growth was mainly contributed by muscle fibre hypertrophy resulting from satellite cell activation, proliferation, differentiation and fusion into the existing fibres. We found that the differential regulation of satellite cell activities might account a lot for divergent muscle growth of broilers and layers. Several growth factors and growth related genes, which play pivotal roles in regulating muscle growth and hypertrophy as strong stimulators or inhibitors of myoblast and satellite cell proliferation and differentiation, were differentially expressed between the two chicken breeds. In addition, we also identified several differentially expressed potential regulators for satellite cell proliferation and differentiation, including LIM-domain containing protein encoding genes *FHL2 *and *CSRP3 *[25,28], as well as skeletal muscle and tendon specific expressed *MUSTN1 *[32].

The microarray data also helped us to investigate the mechanisms of metabolic rate difference giving rise to divergent muscle growth and hypertrophy between broilers and layers. In our microarray data, many broiler and layer differentially expressed genes were classified into functional categories involved in metabolic processes. Genes encoding some glucose metabolic related enzymes (*PDHX*, *ALDH1A2*, *AER61*, *AOX1*) and fatty acid transportation and utilization related proteins (*FABP4 *and *FABP6*) expressed higher in broilers than in layers, whereas a fat break-down related enzyme thiolesterase B expressed higher in layers, suggested that different metabolic regulatory networks are indispensable for the differential growth rates between broilers and layers. The expression of 3-oxoacid CoA transferase 1 (*OXCT1*), a key enzyme of ketone body utilization, was higher in broilers than in layers. Skeletal muscle is one of the main target organs for ketone utilization. Previously published work showed that *OXCT1 *expression was increased during anaerobisis [47]. Since the breast muscles of broilers contain more glycolytic IIB myofibres than those of layers, broiler muscle cells are likely to prefer to utilize ketone bodies rather than fatty acids for aerobic metabolism. Feng Y *et al *found that OXCT1 is one of the most abundantly expressed proteins in human hepatocarcinoma cell line SMMC-7721 [48], indicated that OXCT1 might also function in cell proliferation and differentiation. Therefore, OXCT1 is not only an enzyme for ketone utilization, but also a cell cycle regulator for controlling satellite cell proliferation and differentiation thereby associated with muscle hypertrophy.

Additionally, muscle growth and hypertrophy can be modulated by the balance between muscle protein synthesis and degradation. Previously published works have shown that chicken strains selected for muscle growth degrade muscle proteins less rapidly than those selected for egg laying [17,18]. In our microarray data, we observed an overall lower expression of protein degradation related genes in broilers than in layers, indicating that more protein accumulation could be one reason for the larger body size of broilers.

It is worth to note that we found many growth or metabolic related genes whose expression pattern matched the growth curves of broilers, layers or both. Since the expression changes of these genes at different developmental stages positively or negatively correlated with the body weight change rate of broilers or layers, these genes might be important regulators of muscle cell growth. Here we only considered genes as independent players, there could be gene groups with additive effect so that the combination of their expression change curves also matched the growth curve of broilers or layers. Due to the intense computation requirements, such gene groups were not identified in the current study.

We also investigated pathways and gene regulatory networks that might involve in the developmental control of muscle divergence. Many metabolic pathways, including lipid metabolism and amino acid metabolism pathways, were found to be enriched of DE genes, thus might be significantly divergent between broilers and layers. As only 8.1% of genes in the chicken genome were mapped to KEGG pathways, most chicken KEGG pathways had very limited number of known genes. As a result, pathways with even a single DE gene could be found as significantly enriched of DE genes. Notably, in this analysis, we only included pathways with no less than 3 known genes, under which criterion 93% of chicken pathways were included.

## Conclusion

In summary, we performed a comprehensive analysis of gene expression differences in skeletal muscle cells between broilers and layers and found hundreds of differentially expressed genes, which provide systematic information for understanding the mechanisms of myogenesis and muscle mass control in chicken. These findings will also shed light on the study of muscle development in other organisms, including some human muscle related diseases. In addition, it is important to note that a substantial proportion of identified differentially expressed genes are functionally unknown cDNAs or ESTs without any annotation. The biological roles of these genes in the regulation of myogenesis await to be clarified with the development of chicken genome annotation and further functional gene studies.

## Methods

### Animals

Meat-type broilers (commercial Arbor Acres) and egg-type layers (White Leghorn) were obtained from China Agricultural University. All chickens were grown under conditions in accordance with the University's Animal Care and Use Committee policy. All chickens were kept in floor pens under the same temperature and lighting conditions. For the first two days after hatch, chickens were kept in floor pens at 35°C under constant light. From the post-hatching third day to 8 weeks, temperature was reduced from 35°C to 18°C in a stepwise manner and the lighting schedule was maintained at a 18 h light and 6 h darkness cycle. Both chicken lines were fed with the same commercial layer diet. All chickens were weighed weekly. At each time point (1 day, 2 weeks, 4 weeks, 6 weeks and 8 weeks), 5 randomly selected male individuals from each group were sacrificed for tissue sampling. *Pectoralis major *muscles were excised and immediately frozen in liquid nitrogen and then stored at -70°C.

### Total RNA preparation and microarray hybridization

Total RNA was isolated from breast muscle cells (Pectoralis major, Pm) using Trizol reagent (Invitrogen) according to the manufacturer's instructions. Twenty microgram total RNA was suspended in RNase-Free water with a final concentration of 1.5 *μ*g/*μ*l. Affymetrix Gene Chip microarray hybridization was carried out according to the Affymetrix Expression Analysis Technical Manual by GeneTech Biotechnology Limited Company (Shanghai, China). Array scanning and data extraction were carried out following the standard protocol.

### Identification of differentially expressed genes

The present genes (represented by probe-sets of Affymetrix Gene Chips^®^) of our microarray data were detected using Affymetrix MAS5 method implemented in Bioconductor packages. Present probe-sets were defined as present in samples of at least one examined time point and were used for all following studies. The expression value of each probe set was normalized and calibrated using the RMA method. Differentially expressed probe-sets between broilers and layers were identified by cutoff of fold-change ≥ 1.5 and p-value < 0.01 in ANOVA (Analysis of Variance) test. To assess the False Discovery Rate (FDR) of the DE gene result, a control expression sets were generated by randomly permuting the expression values of all arrays for each gene 500 times. As a result, the estimated FDR of our approach for detecting DE genes was 0.02%; such high specificity would mainly result from the relative large sample size (N = 30).

### Gene Ontology enrichment analysis and visualization

Gene Ontology enrichment analysis was performed using GOEAST software toolkit [49]. The significance level of GO term enrichment was set as FDR-adjusted p-value smaller than 0.1 by the Yekutieli method.

### Determination of gene expression traits (QTE) for chicken muscle growth

To model chicken muscle growth rate, the body mass of individual broiler and layer chickens was measured at different time points, ranging from 0 day to 7 weeks after hatch, with ~7 day intervals. Body mass was first fitted as a function of time after hatch, using the following logistic regression model:

$$W(t)=\frac{\bar{W}}{1+\left(\frac{\bar{W}}{W_{0}}-1\right)e^{-r(t-t_{0})}},$$

where *W*(*t*) is the body mass, *W*_0 _is the initial body mass immediately after hatch (at time *t*_0_), $\bar{W}$ is the maximum body weight limit, and *r *is the absolute growth rate, both $\bar{W}$ and *r *are unknown parameters. The average initial body mass of each chicken line was used as *W*_0_, $\bar{W}$ and *r *were calculated independently for broilers and layers by Gauss-Newton nonlinear least square regression. Then the absolute Growth Rate (GR) and the Natural Increase Rate (NIR) of chicken body mass could be calculated by

$$\begin{array}{l} GR(t)=\frac{dW}{dt}=\frac{r\bar{W}(\bar{W}/W_{0}-1)e^{-r(t-t_{0})}}{{\left[1+(\bar{W}/W_{0}-1)e^{-r(t-t_{0})}\right]}^{2}}\text{ and}\\ NIR(t)=\frac{1}{W}\frac{dW}{dt}=r\left[1-\frac{1}{1+(\bar{W}/W_{0}-1)e^{-r(t-t_{0})}}\right]. \end{array}$$

By the later function, the NIR of broilers and layers at each examined time point was obtained; these data were then used in company with the microarray expression data to identify significant QTE genes of broilers and layers by fitting the following simple linear model to each present gene *i*

*NIR*_*ij *_= β_*i*_*G*_*ij *_+ α_*i *_+ ε_*i*_,

where *NIR*_*ij *_and *G*_*ij *_is the NIR and log expression value for gene *i*at time point *j*, respectively; significant QTE genes were finally determined as those with significant β_ι _≠ 0 (p < 0.05, t-test).

### Analysis of pathways and regulatory networks for chicken muscle development

KEGG pathway [42-44] information was used in this analysis. Probeset IDs of each category were first mapped to NCBI Entrez gene IDs according to the Affymetrix Chicken Array annotation file, and then were mapped to KEGG gene IDs according to the KEGG gene cross-reference file. Pathways that significantly enriched of DE or QTE genes were identified by hypergeometric test using R packages (p < 0.1, FDR adjusted). Small pathways with < 3 known chicken genes were discarded. Graphical pathway maps were downloaded from the KEGG FTP server, DE genes and QTE genes were then high-lighted in them according to the coordinate description in XML files at the KEGG FTP server, using Perl GD, XML::Parser and XML::LibXML modules.

### Quantitative real time RT-PCR

PCR analyses were performed using the ABI Prism 7500 System. Real-time quantification was employed using SYBR Green PCR Master Mix (ABI). PCR primers were designed using AB PRISM Primer Express 2.0 software. To avoid amplification of contaminated genomic DNA, one of the two primers was placed at or just outside of the exon/exon junction. BLASTN searches were performed to confirm the gene specificity of the primer sequences. All primers for qRT-PCR were listed in Additional file 3. qRT-PCR was performed in triplicates with standard deviations of threshold cycle (CT) values not exceeding 0.5. After a general reverse transcription reaction, PCR analyses were performed in the 20 *μ*l amplification reactions containing 10 *μ*l of SYBR Green PCR Master Mix, 20 ng cDNA and 0.5 *μ*M of each primer at the following conditions: 95°C for 10 minutes for 1 cycle, 40 cycles at 95°C for 15 seconds and then at 60°C for 1 minute. Because postnatal 2 weeks to 4 weeks is the period that most divergent growth rate occur between broilers and layers, only RNA samples from postnatal 2 weeks and 4 weeks were used in the qRT-PCR experiment.

## Abbreviations

(IGF): Insulin-like growth factor; (PI3K): phosphatidylinositol 3-kinase; (mTOR): mammalian target of rapamycin; (AMPK): AMP-activated protein kinase; (ERK1/2): extracellular signal regulated kinase 1/2; (NF-kappa B): nuclear factor-kappa B; (PKC): protein kinase C; (*SS*): somatostatin; (*GHR*): growth hormone receptor; (*MYH7B*): myosin heavy chain 7B; (*TNNI1*): troponin I type 1; (*JNK1*): Janus kinase 1; (*P38*): serine/threonine kinase 38 like; (*INCENP*): inner centromere protein antigens; (*NDE1*): nudE nuclear distribution gene E homolog 1; (*MUSTN1*): musculoskeletal embryonic nuclear protein 1; (*CSRP3*): cysteine and glycine-rich protein 3; (*FHL2*): four and a half LIM domains 2; (*FGFR2*): fibroblast growth factor receptor 2; (*FGF16*): fibroblast growth factor 16; (*HS6ST2*): heparin sulphate 6-O-sulfotransferase 2; (HSPGs): heparan sulfate proteoglycans; (*MB*): myoglobin; (*CES1*): thiolesterase B; (*PDHX*): pyruvate dehydrogenase complex, component X; (*ALDH1A2*): aldehyde dehydrogenase 1 family, member A2; (*AER61*): glycosyltransferase AER61; (*AOX1*): aldehyde oxidase 1; (*FABP4*): fatty acid binding protein 4; (*FABP6*): intestinal 15 kda protein; (ELOVL 6): homolog, elongation of long chain fatty acids; (*PDK4*): pyruvate dehydrogenase kinase 4; (*OXCT1*): 3-oxoacid CoA transferase 1; (*COMT*): Catechol-O- -methyltransferase; (*FBXO22*): F-box proteins 22; (*FBXO30*): F-box proteins 30, (*UCH-L1*): ubiquitin carboxyl-terminal hydrolase isozyme L1; (*HERC4*): hect domain and RLD 4; (*HERC5*): hect domain and RLD 5; (*RNF12*): ring finger protein 12; (*AER61*): glycosyltransferase; (*FGF1*): fibroblast growth factor 1 (acidic); (*DKK3*): dickkopf homolog 3; (*EDNRA*): endothelin receptor type A; (*EDNRB*): endothelin receptor type B; (*PTCHD1*): patched domain containing 1; (*AER61*): glycosyltransferase; (LARGE): like-glycosyltransferase; (*ACTN3*): actinin, alpha3; (*MYBPC3*): myosin binding protein C, cardiac.

## Authors' contributions

YZ carried out the experimental design and performed the experiments and drafted the manuscript. QZ and YC carried out all the bioinformatics analysis and participated in the manuscript preparation. NY carried out animal care and tissue sampling. DZ and X-JW conceived of the study, and participated in its design and coordination and helped to draft the manuscript. All authors read and approved the final manuscript.

## Supplementary Material

Additional file 1 **Growth rates of broiler and layer chickens**. This figure presents the growth rates of broiler and layer chickens. The absolute growth rates (unit: Δg) and the Natural Growth Rates (unit: Δg/g) of broiler and layer chickens are shown as a function of days after hatch (DAH). Red lines, broilers; blue lines, layers; solid lines, absolute growth rate; dashed lines, Natural Growth Rates (NIR). Click here for file

Additional file 2 **Annotation and expression values of the 543 differentially expressed genes between broilers and layers**. This file provides the probe set annotations and expression values of the 543 differentially expressed genes at all examined time points. Click here for file

Additional file 3 **List differentially expressed genes between broilers and layers validated by qRT-PCR**. This file includes the information of differentially expressed genes confirmed by qRT-PCR. The primer sequences used for qRT-PCR are also included. Click here for file

Additional file 4 **Enriched GO terms among the 543 differentially expressed probe sets between broilers and layers**. This file includes the list of enriched GO terms among the 543 differentially expressed probe sets between broilers and layers, as well as the enrichment degree of each GO term. Click here for file

Additional file 5 **Differentially expressed genes in the chicken tyrosine metabolism pathway**. This file presents the differentially expressed genes in chicken tyrosine metabolism pathway. The differentially expressed genes within the tyrosine metabolic pathway are highlighted by red ellipses. Click here for file

Additional file 6 **List of genes with expression patterns correlated with the growth rates of broilers and/or layers (QTE genes)**. This file includes the list of genes with expression patterns correlated with growth rates of broilers and/or layers. The probe information, annotation and expression values of each gene are included. Click here for file

Additional file 7 **Comparison of enriched GOBP Terms for DE genes vs. common-QTE genes**. This file presents the enriched biology process GO terms of the differentially expression genes and the common QTE genes between broilers and layers. Red boxes, enriched GO BP terms for DE genes; Green boxes, enriched GO BP terms for common-QTE genes; Yellow, Shared GO BP terms for both DE-genes and common-QTE genes. The colour saturation degree is positively correlated with the significance of enrichment. Click here for file

Additional file 8 **Gene Ontology analysis of broiler specific QTE genes**. Enriched biological process GO terms are highlighted by yellow; the colour saturation degree is positively correlated with the significance of enrichment. Click here for file

Additional file 9 **Gene Ontology analysis of layer specific QTE genes**. Enriched biological process GO terms are highlighted by yellow; the colour saturation degree is positively correlated with the significance of enrichment. Click here for file

Additional file 10 **Enriched KEGG pathways among the 543 differentially expressed genes between broilers and layers**. This file lists the enriched KEGG pathways among the 543 differentially expressed genes between broilers and layers. Pathways with FDR less than 0.1 are included. Click here for file
