# Supplementary figures and images for: Systematic identification of genes involved in divergent skeletal muscle growth rates of broiler and layer chickens
Source: BMC Genomics. 2009 Feb 22;10:87. doi: 10.1186/1471-2164-10-87 (PMC2656524; doi:10.1186/1471-2164-10-87)

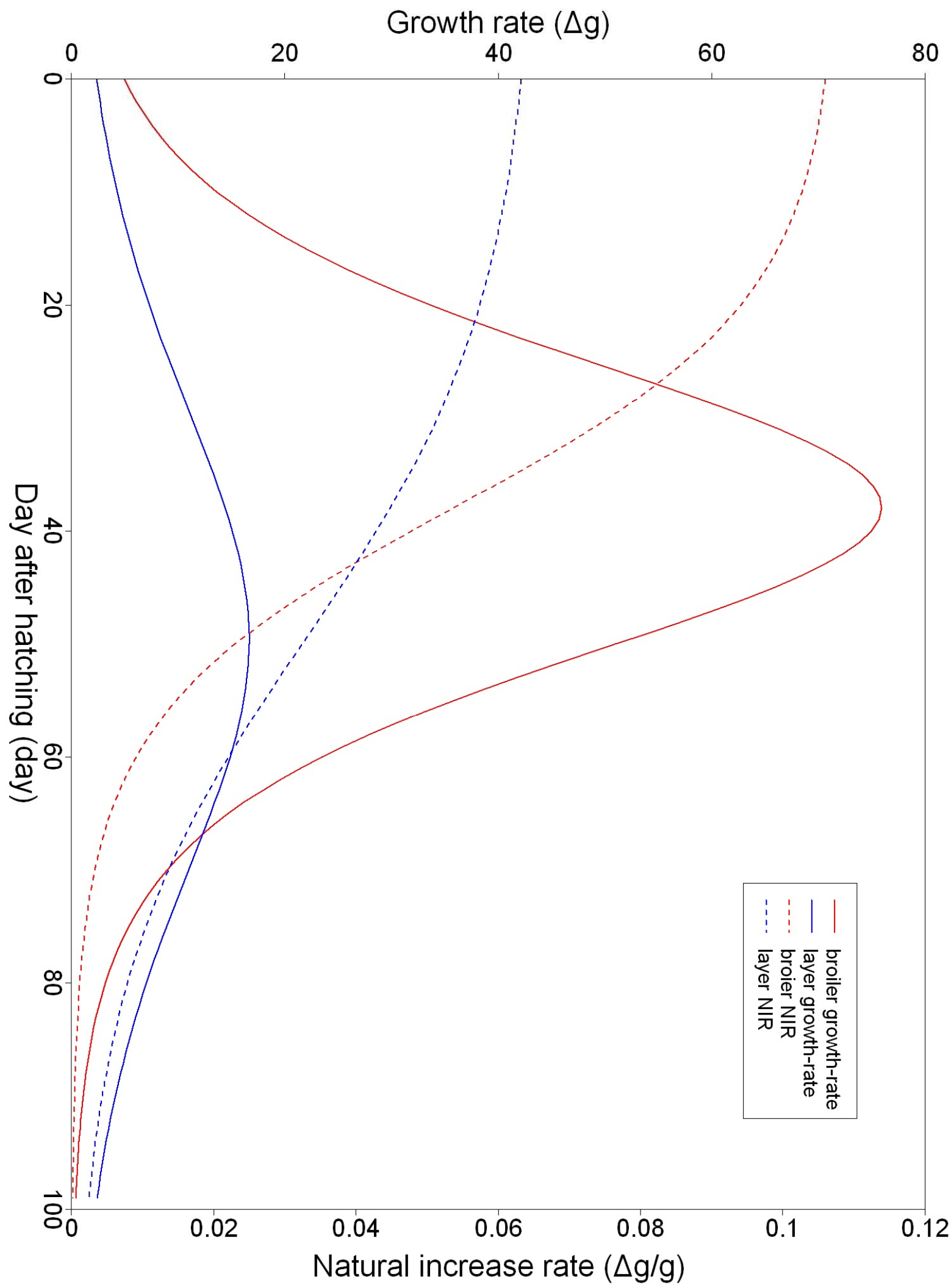

Supplement: Additional file 1 — Growth rates of broiler and layer chickens. This figure presents the growth rates of broiler and layer chickens. The absolute growth rates (unit: Δg) and the Natural Growth Rates (unit: Δg/g) of broiler and layer chickens are shown as a function of days after hatch (DAH). Red lines, broilers; blue lines, layers; solid lines, absolute growth rate; dashed lines, Natural Growth Rates (NIR). [file 1471-2164-10-87-S1.pdf]

## DDT degradation

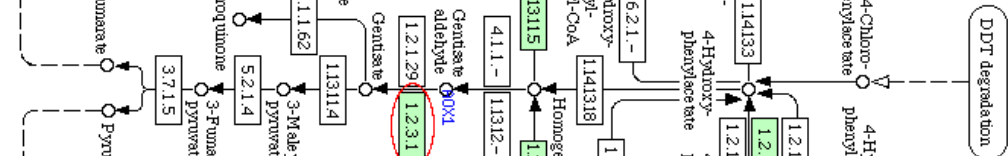

Supplement: Additional file 5 — Differentially expressed genes in the chicken tyrosine metabolism pathway. This file presents the differentially expressed genes in chicken tyrosine metabolism pathway. The differentially expressed genes within the tyrosine metabolic pathway are highlighted by red ellipses. [file 1471-2164-10-87-S5.pdf]

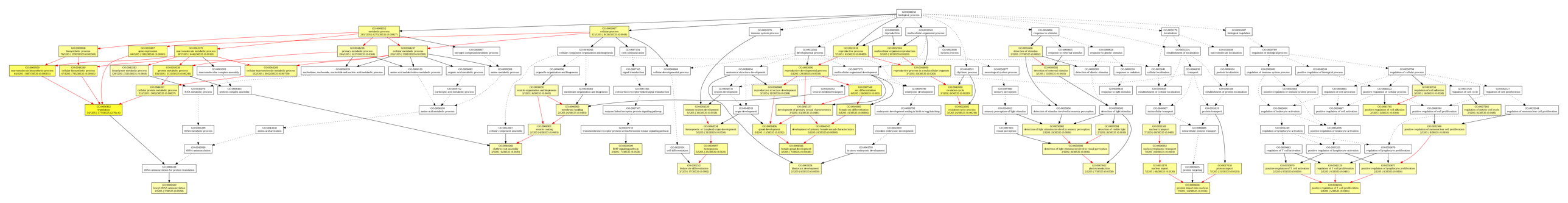

Supplement: Additional file 8 — Gene Ontology analysis of broiler specific QTE genes. Enriched biological process GO terms are highlighted by yellow; the colour saturation degree is positively correlated with the significance of enrichment. [file 1471-2164-10-87-S8.pdf]

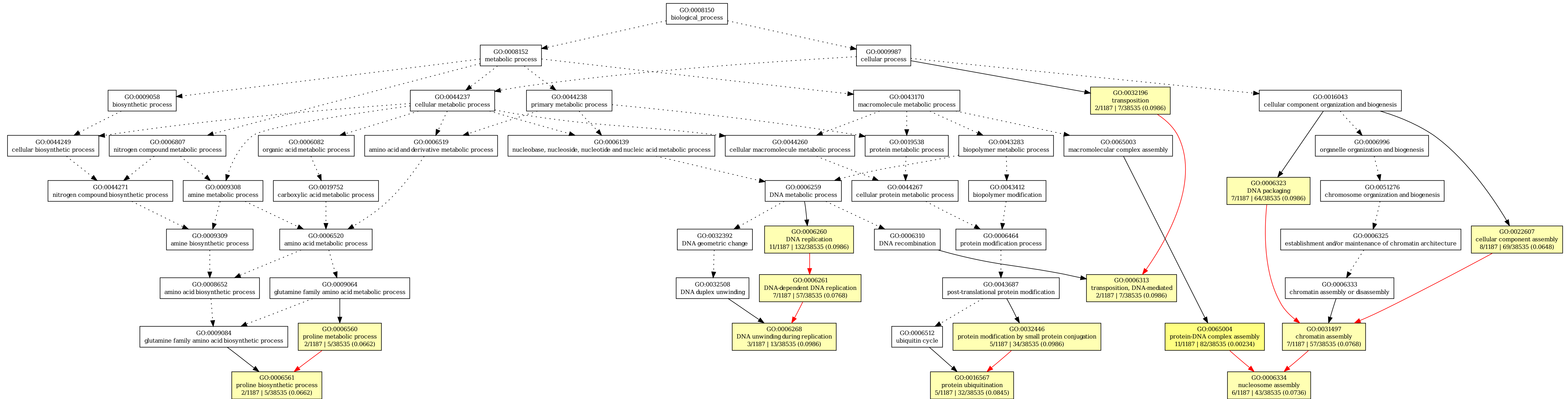

Supplement: Additional file 9 — Gene Ontology analysis of layer specific QTE genes. Enriched biological process GO terms are highlighted by yellow; the colour saturation degree is positively correlated with the significance of enrichment. [file 1471-2164-10-87-S9.pdf]
