# Supplementary material for: Systematic identification of genes involved in divergent skeletal muscle growth rates of broiler and layer chickens
Source: BMC Genomics. 2009 Feb 22;10:87. doi: 10.1186/1471-2164-10-87 (PMC2656524; doi:10.1186/1471-2164-10-87)

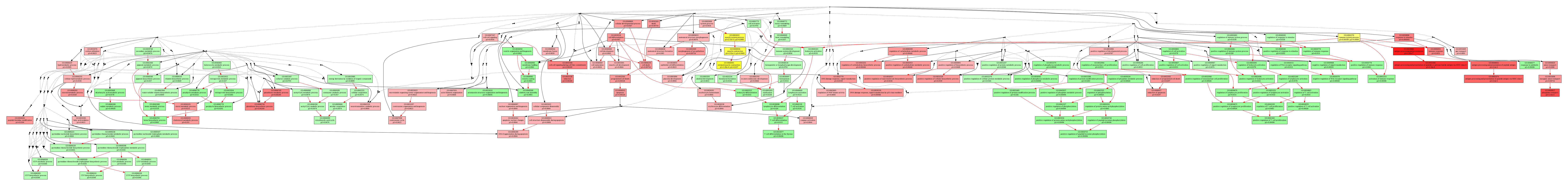

Supplement: Additional file 7 — Comparison of enriched GOBP Terms for DE genes vs. common-QTE genes. This file presents the enriched biology process GO terms of the differentially expression genes and the common QTE genes between broilers and layers. Red boxes, enriched GO BP terms for DE genes; Green boxes, enriched GO BP terms for common-QTE genes; Yellow, Shared GO BP terms for both DE-genes and common-QTE genes. The colour saturation degree is positively correlated with the significance of enrichment. [file 1471-2164-10-87-S7.pdf]
